# Dietary effects on breast cancer molecular subtypes, a 1:2 paired case–control study

**DOI:** 10.1002/fsn3.1866

**Published:** 2020-08-31

**Authors:** Guohai Yuan, Jingjing Zhang, Yi Ren, Wei Ding, Yan Du, Lu Zhang, Jihong Shao

**Affiliations:** ^1^ Department of Nutrition School of Public Health Xuzhou Medical University Xuzhou China; ^2^ Breast Surgery Xuzhou Cancer Hospital Xuzhou China; ^3^ Thyroid Breast Surgery The Affiliated Hospital of Xuzhou Medical University Xuzhou China

**Keywords:** breast cancer, case–control study, dietary factor, molecular subtypes

## Abstract

To explore the associations between dietary factors and breast cancer (BC) molecular subtypes. The retrospective cases were confirmed by pathological diagnosis with breast cancer were gathered in two major hospitals in Xuzhou city, China, from 2015 to 2016. These cases were classified by the meeting standard of 13th St Gallen: luminal A, luminal B, Her‐2 overexpression, and triple‐negative breast cancer (TNBC) subtypes. A 1:2 paired retrospective case–control study with 210 cases and 420 controls was conducted to evaluate individual dietary intake, by food frequency questionnaire (FFQ) and estimate odds ratios (*OR*s), by the Cox regression model. For overall breast cancer patients, the more frequency of red meat (*OR* = 1.002, *95% CI* = 1.001–1.004) and salted food (*OR* = 1.003, *95% CI* = 1.001–1.005) were statistically significantly associated with a greater risk of breast cancer. Beans (*OR* = 0.997, *95% CI* = 0.995–0.999), white meat (*OR* = 0.993, *95% CI* = 0.989–0.997), aquatic products (*OR* = 0.990, *95% CI* = 0.984–0.996), vegetables (*OR* = 0.999, *95% CI* = 0.999–0.999), fruit (*OR* = 0.998, *95% CI* = 0.997–0.999), and green tea (*OR* = 0.997, *95% CI* = 0.994–0.999) were significantly associated with a lower risk of breast cancer. For luminal breast cancer patients, beans (*OR* = 0.997, *95% CI* = 0.994–0.999), white meat (*OR* = 0.992, *95% CI* = 0.987–0.997), green tea (*OR* = 0.995, *95% CI* = 0.991–0.999), and milk (*OR* = 0.998, *95% CI* = 0.996–0.999) were protective factors. While for nonluminal breast cancer, red meat was not included in the equation, and beans (*OR* = 0.989, *95% CI* = 0.981–0.997), white meat (*OR* = 0.989, *95% CI* = 0.981–0.998), vegetables (*OR* = 0.998, *95% CI* = 0.997–0.999), and milk (*OR* = 0.994, *95% CI* = 0.989–0.999) still showed a significantly reduced risk of nonluminal breast cancer. Different dietary factors revealed different effects on the etiology of breast cancer. Red meat may be a specific risk factor for luminal‐type breast cancer.

## Graphical abstract

The paper was to explore the associations between dietary factors and breast cancer molecular subtypes by food frequency questionnaire. The result showed that different dietary factors revealed different effects on the etiology of breast cancer, and red meat may be a specific risk factor for luminal‐type breast cancer.

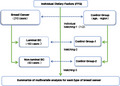

## 1 INTRODUCTION

Gene expression studies since 2000 have revealed that breast cancer is a highly heterogeneous disease, which has led to molecular classification of breast carcinomas (Perou et al., 2000). Recent studies have indicated that the molecular subtypes of breast cancer play a pivotal role in the recurrence, metastasis, and survival condition of breast cancer (Jones et al., 2016; Tsoutsou, Vozenin, Durham, & Bourhis, 2017; Vasconcelos et al., 2016; Wu et al., 2016). The unique system of each type (Goldhirsch et al., 2011, 2013) suggests that each type of breast cancer may also have specific risk factors. Current epidemiological studies have shown that breast cancer is related mainly to reproductive factors (menstrual history, birth history, and breastfeeding history, *etc*.), and genetic factors and lifestyle (diet, spirit and exercise, *etc*.); however, the associations between these factors and each molecular subtype of breast cancer are not distinct (Brouckaert et al., 2018; Tamimi et al., 2012; Zhang et al., 2019). In recent years, reproductive factors showed etiological differences in each subtype of breast cancer, but associations with other risk factors were still inconsistent, including dietary risk factors (Gaudet et al., 2018; Lambertini et al., 2016). The present study explores and validates the differences in dietary risk factors for molecular types of breast cancer.

## 2 MATERIAL AND METHODS

### 2.1 Subjects

The 1:2 case–control study was taken in overall breast cancer patients. From 1 June 2015 to 30 Oct 2016, a total of 210 female patients of confirmed breast cancer with integrated information were gathered through the electronic medical record systems from two major hospitals in Xuzhou City, China. The records of the control group of 420 people matched by age and area were taken from the national public service project of “Screening for Cervical Cancer and Breast Cancer” either in the local hospital or community. For the further analysis, the 210 breast cancer patients were divided into luminal and nonluminal‐type breast cancer according to molecular subtype, including 150 pairs of 1:2 matched luminal cases and 60 pairs of 1:2 matched nonluminal cases. The 1:2 paired case–control study was also taken in luminal and nonluminal‐type breast cancer, respectively.

### 2.2 Classification of breast cancer subtypes

The detection of receptors was performed by pathologists in hospital laboratories. The expression of ER and PR were graded according to the percentage of positive cells in the tumor cells: <1% was negative, ≥1% was positive. For the Ki‐67 receptor, ≥14% was considered as positive. According to the Breast Cancer Her‐2 Test Guideline, the Her‐2 receptor was defined as negative with the result of − or +, while her‐2 +++ was positive. For the intermediate state Her‐2 ++, fluorescence in situ hybridization (FISH) was required to confirm the her‐2 receptor further. Moreover, Her‐2 ++ without FISH test was considered by default to be negative.

The patients were classified by molecular subtypes according to the definition of the 11th St Gallen: luminal A subtype (ER+ and/or PR+, Her‐2‐, Ki‐67 < 14%), luminal B subtype (ER+ and/or PR+, Her‐2+ or Ki‐67 ≥ 14%), Her‐2 overexpression subtype (ER‐, PR‐, Her‐2 +), and TNBC (ER‐, PR‐, Her‐2 −). Due to the similar etiological characteristics between luminal A and luminal B breast cancer, they were combined as luminal type, and the other two types were nonluminal type.

### 2.3 Data collection and quality control

The questionnaire collected fundamental and clinicopathological profiles included age at diagnosis, region, menstrual history, reproductive history, family history, TNM status, histological type, and expressions of ER, PR, Her‐2, and Ki67. Results of Her‐2 fluorescence in situ hybridization (FISH) testing were also available. In addition, we took a national semi‐quantitative food frequency questionnaire method (FFQ) to reflect individual long‐term dietary patterns. Referring to the results of the current dietary studies of breast cancer, the food was sorted into beans, red meat, white meat, aquatic products, vegetables, and fruits. The face‐to‐face survey was completed by graduate students trained in uniform standards at hospital or community health center for reducing information bias. To correct the dietary questionnaires for measurement errors, measuring aid was used to investigate the frequency and amount of intake of these foods in the past year. For analysis, the frequency was finally converted into times of consumption per year.

### 2.4 Statistical analysis

Epidata version 3.1 and SPSS version 16.0 were used to establish and analyze the database. Measurement data were expressed by ($\bar{x}\pm s$). The Cox regression model was used to analyze the influencing factors of breast cancer and its subtypes. The test level was 0.05. The study protocol was approved by removed for blind peer review. This study was prepared based on strengthening the reporting of observational studies in epidemiology (STROBE) statement for case–control studies. The study was approved by the Institutional Review Board of the Affiliated Hospital of Xuzhou Medical University (XYFY2015‐KL071‐01). Written informed consent was obtained from all adult subjects.

## 3 RESULTS

### 3.1 Study population characteristics

A total of 210 breast cancer cases and 420 control subjects matched by age and area were included in the study. Table 1 shows the epidemiological and physiological characteristics of the study population of breast cancer and each type.

**Table 1:** Epidemiological and physiological characteristics of breast cancer and each type

Variables	Overall cases (*n* = 210)	Luminal cases (*n* = 150)	Nonluminal cases (*n* = 60)
Age at diagnosis (mean, *SD*)	50.5 (8.5)	49.95 (8.6)	51.9 (8.3)
BMI (mean, *SD*)	23.9 (3.0)	24.1 (3.0)	23.6 (2.9)
WHR (mean, *SD*)	0.9 (0.1)	0.9 (0.1)	0.9 (0.1)
Education			
Below college (*n*, %)	192 (91.4%)	136(90.7%)	56(93.3%)
College and above (*n*, %)	18 (8.6%)	14(9.3%)	4(6.7%)
Age at menarche (mean, *SD*)	15.1 (2.2)	15.1 (2.2)	15.1 (2.4)
Premenopausal (*n*, %)	119 (56.7%)	91 (60.7%)	28 (46.7%)
Drinking (*n*, %)	17 (8.1%)	10 (6.7%)	7 (11.7%)
Smoking (*n*, %)	2 (1.0%)	2 (1.3%)	0 (0%)
Passive smoking (*n*, %)	120 (57.1%)	83 (55.3%)	37 (61.7%)
Family history of cancer (*n*, %)	41 (19.5%)	33 (22.0%)	8 (13.3%)

### 3.2 Breast cancer risk by dietary factors, overall, and by subtype

Multivariate analysis of breast cancer showed that adjusting for other factors such as waist‐to‐hip ratio (WHR), family history of cancer and passive smoking, etc., annual intake of red meat (*OR* = 1.002, *95% CI* = 1.001–1.004), and salted products (*OR* = 1.003, *95% CI* = 1.001–1.005) were determined to be independent risk factors for breast cancer versus control. Annual frequency intake of beans (*OR* = 0.997, *95% CI* = 0.995–0.999), white meat (*OR* = 0.993, *95% CI* = 0.989–0.997), aquatic products (*OR* = 0.990, *95% CI* = 0.984–0.996), fruit (*OR* = 0.998, *95% CI* = 0.997–0.999), green tea (*OR* = 0.997, *95% CI* = 0.994–0.999), and vegetables (*OR* = 0.999, *95% CI* = 0.999–0.999) were statistically significantly associated with a lower risk for breast cancer (Tables 2, 3).

**Table 2:** Multivariate analysis for overall breast cancer and each type (Cases versus Normal)

Variables	Overall cases (*n* = 210)		Luminal cases (*n* = 150)		Nonluminal cases (*n* = 60)	
	ORs	95% CI	ORs	95% CI	ORs	95% CI
Beans	0.997	0.995–0.999	0.997	0.994–0.999	0.989	0.981–0.997
Red meat	1.002	1.001–1.004	1.003	1.001–1.005	‐	‐
White meat	0.993	0.989–0.997	0.992	0.987–0.997	0.989	0.981–0.998
Aquatic products	0.990	0.984–0.996	‐	‐	‐	‐
Vegetables	0.999	0.999–0.999	‐	‐	0.998	0.997–0.999
Fruit	0.998	0.997–0.999	‐	‐	‐	‐
Salted products	1.003	1.001–1.005	1.002	1.001–1.004	1.006	1.000–1.011
Tea	0.997	0.994–0.999	0.995	0.991–0.999	‐	‐
Milk	‐	‐	0.998	0.996–0.999	0.994	0.989–0.999

‐, the factor was not in the regression model.

**Table 3:** Summarize of analysis for each type of breast cancer

Dietary factors	Breast cancer	Luminal breast cancer	Nonluminal breast cancer
Beans	−	−	−
Red meat	+	+	X
White meat	−	−	−
Aquatic products	−	X	X
Vegetables	−	X	−
Fruit	−	X	X
Salted products	+	+	+
Green tea	−	−	X
Milk	X	−	−

+, risk factors, −, protective factors, X, indifferent.

Multivariate analysis for luminal breast cancer and paired control showed that in the combination of WHR, family history of cancer and physical exercise, etc., annual frequency intake of red meat (*OR* = 1.003, *95% CI* = 1.001–1.005) and salted products (*OR* = 1.002, *95% CI* = 1.001–1.004) were again determined to be significant risk factors. Annual intake of beans (*OR* = 0.997, *95% CI* = 0.994–0.999), white meat (*OR* = 0.992, *95% CI* = 0.987–0.997), green tea (*OR* = 0.995, *95% CI* = 0.991–0.999), and milk (*OR* = 0.998, *95% CI* = 0.996–0.999) were seen to result in a reduced risk for luminal breast cancer (Table 2).

Multivariate analysis for nonluminal breast cancer showed that adjusting for other risk factors, the annual frequency intake of beans (*OR* = 0.989, *95% CI* = 0.981–0.997), white meat (*OR* = 0.989, *95% CI* = 0.981–0.998), vegetables (*OR* = 0.998, *95% CI* = 0.997–0.999), and milk (*OR* = 0.994, *95% CI* = 0.989–0.999) were significantly protective factors against nonluminal breast cancer (Table 2).

## 4 DISCUSSION

Both prospective studies and retrospective studies have confirmed that intake of beans plays a significant protective role in preventing breast cancer (Iwasaki et al., 2009; Ward et al., 2010; Wu, Koh, Wang, Lee, & Yu, 2008). Isoflavone is a vital ingredient in beans, which is similar structurally to the natural estrogen secreted in humans. Isoflavone can bind to estrogen receptors in the body and produce a double effect, weak estrogen‐like, and antagonize estrogen effect, in safeguarding women (Hooper, Madhavan, Tice, Leinster, & Cassidy, 2010; Li, Yuan, Meeran, & Tollefsbol, 2010; Limer & Speirs, 2004). This study showed the similar effect of beans on breast cancer and on each subtype, which is consistent with previous studies. Red meat (meat that appeared crimson before cooking, such as pork, beef, and lamb) was rich in total fat, especially saturated fatty acids. White meat refers to meat with fine muscles, few saturated fatty acids and ample unsaturated fatty acid, such as chicken, duck, and goose. The fat in meat was speculated to play an important role in the etiology of breast cancer. It is well known that the imbalance between estrogen and progesterone is an important cause of breast cancer. The fat provided not only storage cells for energy, but also promoted the production of estrogen, stimulating the proliferation of normal breast tissue and mammary epithelial cell, and thus increasing the risk of BC. By meta‐analysis method, Yand, Lü, Zhou, & Ye, (2016) showed that a high intake of red meat increased the risk of breast cancer by 8% compared with low intake. Meanwhile, it was found that meat intake based on poultry or freshwater fish could reduce the morbidity of breast cancer (Zhang et al., 2009). Sieri et al., (2008) in the European Cancer and Nutrition Cohort Study found that saturated fatty acids could promote the occurrence of breast cancer. The results of this study are consistent with those results. Also, this study indicated that a higher intake of salted food was associated with more risk of breast cancer, and for each subtype.

In this study, red and white meat had different effects on luminal and nonluminal‐type breast cancers. In a cohort study of 337,327 members, Sieri et al., (2014) found that high total fat and saturated fat levels were associated with ER+/PR+‐positive breast cancer, but not ER‐PR‐negative breast cancer. This study shows that red meat saturated fatty acid is involved in the etiology of luminal BC, but not nonluminal‐type breast cancer, which was consistent with the effect of WHR on luminal BC in this study. Red meat may be a specific risk factor for luminal‐type breast cancer, due to the saturated fat. However, the specific mechanism of action is unclear and will require further study. Cox regression analysis showed that vegetables and fruits could reduce the risk of breast cancer, whereas no similar effects on all types of breast cancer. This difference may be due to insufficient sample size or to imprecise division of subtypes of vegetables.

In brief, different dietary factors could have different and specific effects on the etiology of breast cancer subtype. Further research will be needed to declare ambiguities of dietary factors for molecular subtypes of breast cancer.

## ETHICS APPROVAL AND CONSENT TO PARTICIPATE

The study was approved by the Institutional Review Board of the Affiliated Hospital of Xuzhou Medical University (XYFY2015‐KL071‐01). Written informed consent was obtained from all adult subjects.

## CONFLICT OF INTEREST

The authors declare no conflict of interest.

## AUTHOR CONTRIBUTION

Guohai Yuan was involved in data collection, analysis of results, and writing the draft manuscript. Jingjing Zhang, Yi Ren, Wei Ding, Yan Du, and Lu Zhang were involved in development of the study design and data analysis. Jihong Shao was involved in development of the analysis of results and contributed to submitting the manuscript. All authors are in agreement with the manuscript and declare that the content has not been published elsewhere.
